# Effect of Sr on Mechanical Properties and Corrosion Behavior of Rolled ZM60 Alloy

**DOI:** 10.3390/ma17246166

**Published:** 2024-12-17

**Authors:** Dongsong Yin, Yuting Zhou, Zhiyuan Liu, Yong Mao, Tianming Han

**Affiliations:** 1 School of Materials Science and Engineering, Guangdong Ocean University, Yangjiang 529500, China; 2 School of Mechanical Engineering, Guangdong Ocean University, Zhanjiang 524088, China; zyt1961715995@163.com; 3 School of Materials Science and Engineering, Heilongjiang University of Science and Technology, Harbin 150022, China; liuzhiyuan0314@163.com (Z.L.); maoyong1996@163.com (Y.M.); 4 School of Materials Science and Engineering, Jiamusi University, Jiamusi 154000, China; 15942020543@163.com

**Keywords:** medical Mg-6Zn-0.5Mn magnesium alloy, strontium, rolling, mechanical properties, corrosion behavior

## Abstract

Mg-6Zn-0.5Mn as a medical magnesium alloy has good biomechanical properties and corrosion resistance, but as a fracture internal-fixation material, its strength, toughness, and corrosion resistance still need to be improved. In this paper, the element Sr, having good biocompatibility, is used as an alloy element. The effects of different Sr contents (0 wt.%, 0.3 wt.%, 0.6 wt.%, 0.9 wt.%, and 1.2 wt.%) on the microstructure, strength, toughness, and corrosion resistance of rolled Mg-6Zn-0.5Mn alloy were studied. The results are as follows. Sr can influence the recrystallization process. When the Sr content is 0.3 wt.% and 0.6 wt.%, the alloy matrix exhibits both non-recrystallized regions and fine recrystallized regions. When the Sr content reaches 0.9 wt.%, the non-recrystallized region decreases significantly, and the fine recrystallized grains develop into equiaxed grains. With the increase of Sr content, the elongation of the alloy decreases. At a content of 0.9 wt.%, the corrosion resistance reaches its optimum value, with an average corrosion rate of 0.75828 mm/y.

## 1. Introduction

Due to its low density, high specific strength, good biocompatibility, and degradability [1], magnesium alloys have shown great application potential in the fields of aerospace, transportation, electronic communication, and biomedicine. In particular, Mg-Zn-Mn series alloys have become a research hotspot due to their excellent comprehensive properties [2,3,4]. In this series of alloys, due to the high electrode potential of Zn, when the element Zn is dissolved into the magnesium alloy, the corrosion potential of the magnesium matrix can be increased, and the corrosion resistance of the alloy can be improved. As an effective strengthening phase, Mn can improve the yield strength of the alloy by forming MnAl_6_ and other phases [5,6,7,8,9]. However, the corrosion resistance of Mg-Zn-Mn alloy still needs to be improved. In order to improve the mechanical properties and corrosion resistance of Mg-Zn-Mn alloys, microalloyed techniques have been extensively studied [10,11,12].

The addition of rare earth elements can effectively improve the mechanical properties and corrosion resistance of magnesium alloys. The addition of trace rare earth elements can refine the grain of the alloy and increase the strength and toughness of the alloy. At the same time, rare earth elements can also improve the corrosion resistance of the alloy, which may be related to the special corrosion products formed on the surface of the alloy [13,14]. Among the many microalloying elements, strontium has similar chemical properties to calcium and is one of the necessary trace elements in the human body. Its maximum solubility in the magnesium matrix is about 0.11 wt.%, which can promote the growth of osteoblasts in the human body [15]. The addition of the element Sr to pure Mg can not only refine the grain size of Mg and enhance the corrosion resistance but also promote the growth of bone tissue [16,17]. Strontium (Sr), as a new alloying element, has little influence on the microstructure and properties of Mg-Zn-Mn alloy. However, its influence on the mechanical properties and corrosion resistance of Mg-Zn-Mn alloy has attracted the attention of researchers. Studies have shown that the addition of strontium is generally able to refine the grain of the alloy and improve the strength and toughness of the alloy through the formation of a Mg-Sr phase [18,19,20,21]. Zhang et al. [22] studied the effect of microalloying the element Sr on the as-cast microstructure and mechanical properties of a Mg-Al-Zn alloy used in vehicle-mounted systems. The results show that the content and shape of the Al_4_Sr, Mg_17_Sr_2_, Mg_32_ (Al, Zn)_49_, and Al_2_Mg_5_Zn_2_ phases in the alloy change with the increase in the Sr content. The yield strength, tensile strength, and elongation of the alloy increased first and then decreased and reached the maximum value when the Sr content was 2%.

Brar et al. [23] studied the microstructure, mechanical properties, and degradation behavior of a Mg-Sr binary alloy and a Mg-Zn-Sr ternary alloy. The results show that Mg-2.0 wt.%Zn-0.5 wt.%Sr and Mg-4.0 wt.%Zn-0.5 wt.%Sr are promising biodegradable alloys with low degradation rates and good mechanical properties. The addition of Zn to Mg-0.5 wt.%Sr alloy increases the yield strength and ultimate tensile strength of the material due to its good solid solution-strengthening and precipitation-strengthening effect. Therefore, it is important to control the microstructure to optimize the mechanical and degradation behavior of the alloy.

The effect of Sr addition on the corrosion resistance of magnesium alloys is still controversial. On the one hand, the metamorphic effect of Sr may help to form a more uniform and dense corrosion product film, thereby slowing down the corrosion rate [24]. On the other hand, the addition of Sr may also change the microstructure of the alloy, which, in turn, affects its corrosion behavior [25,26]. However, there is a lack of systematic studies on the specific effects of Sr on the corrosion rate of Mg-Zn-Mn alloys.

The deformation process has a significant effect on the mechanical properties and corrosion behavior of Mg-Zn-Mn alloys. Through the extrusion process, the microstructure of the alloy can be significantly improved, thereby improving its mechanical properties and corrosion resistance [27].

Feng et al. [28] studied the effect of corrosion of as-cast and extruded Mg-Sm-Zn-Zr alloys in a NaCl solution. Hot extrusion deforms the relatively large mesh Mg_41_Sm_5_ phase into uniformly dispersed fine particles, effectively alleviating local corrosion and forming a uniform protective film on the alloy surface. Compared with the as-cast alloy, the corrosion resistance of the extruded Mg-Sm-Zn-Zr alloy increased by three times, which was better than that of the AZ91 magnesium alloy. Shahriar et al. [29] found that the hot-rolling process helps to improve the corrosion resistance of Mg-1Ca alloy in simulated body fluid (SBF). When the rolling temperature is 330 °C, Mg-1Ca alloy with 60% deformation can be used as a suitable bone implant material to achieve higher corrosion resistance.

In conclusion, the addition of the element Sr provides an effective way to improve the mechanical properties and corrosion resistance of ZM60 magnesium alloy in the rolled state. By precisely controlling the amount of Sr addition and the deformation process, the properties of the alloys can be further optimized to meet the needs of specific application environments. Future studies will further explore the mechanism of Sr elements in magnesium alloys and how to synergetically interact with other alloy elements to achieve a comprehensive improvement in the properties of magnesium alloys.

## 2. Materials and Methods

### 2.1. Preparation of Experimental Materials

In the experiment, ZM60 (Mg-6Zn-0.5Mn) magnesium alloy was used as the matrix. In the preparation of alloy samples, the alloy with a total mass of 1000 g was prepared according to the formula of Mg-6Zn-0.5Mn-xwt.%Sr (x = 0, 0.3, 0.6, 0.9, and 1.2). The materials(Hunan Rare Earth Metal and Material Institute, China) used were pure magnesium (99.99%), zinc particles (99.99%), Mg-10%Mn, and Mg-20%Sr intermediate alloys. Before alloy melting, all experimental raw materials, covering agents, molds coated with zinc oxide, slag picking spoons, and other tools were put into a drying oven preheated to 200 °C for drying and preheating.

The melting process was carried out in a well-type resistance furnace, and the specific steps were as follows. First, the dried magnesium ingots with a purity of 99.99% were put into a graphite crucible, and the surface was evenly sprinkled with a covering agent. When the furnace temperature was raised to 750 °C, the protective layer was removed, and the intermediate alloy was added in turn, with 10 min of insulation required for each addition to ensure complete melting of the alloy. After melting was completed, the mixture was stirred evenly using a stirring tool, and then, the mixture was kept warm and left for 15 min to remove surface scum. Finally, the molten metal was poured uniformly into a metal mold preheated to 200 °C, and the mold and alloy were cooled to room temperature before being removed for subsequent use.

A ZK-WS2A5 horizontal double roller mill was used for rolling. The roll was preheated to 200 °C before rolling. The samples were preheated to 300 °C in a SX2-10-12N (Shanghai Lichen Instrument Technology Co., Ltd., Shanghai, China) heat treatment furnace and held for 10 min. The rolling speed was set at 2 r/min. The amount of each rolling was 10%, and the annealing treatment was carried out at 300 °C for 10 min between each pass. Plates with a thickness of 10 mm were rolled to 1 mm.

### 2.2. Microstructural Characterization

The morphology was analyzed using a Zeiss Axio Lab.A1 Zeiss metallographic microscope (Carl Zeiss AG, Oberkochen, Germany). A scanning electron microscope (SEM) of SU5000 (Hitachi High-Tech Corporation, Tokyo, Japan) was used to analyze the surface morphology, second-phase distribution, fracture morphology, and corrosion morphology of the alloy. At the same time, the energy spectrum analyzer (EDS), combined with SEM, was used to analyze the microzone components in detail. The alloy was corroded with picric (5 g) + glacial acetic (5 mL) + ethanol (100 mL) + distilled water (10 mL). In addition, to detect the phase composition of the alloy, a Cu-Kα target X-ray diffractometer (XRD) DX-2700B model (Dandong Haoyuan Instrument Co., Ltd., Dandong, China) was used with a scanning range of 2θ = 15° to 80°, a scanning speed of 4°/min, and an operating voltage of 30 KV.

### 2.3. Tensile Tests

**Figure 1:**
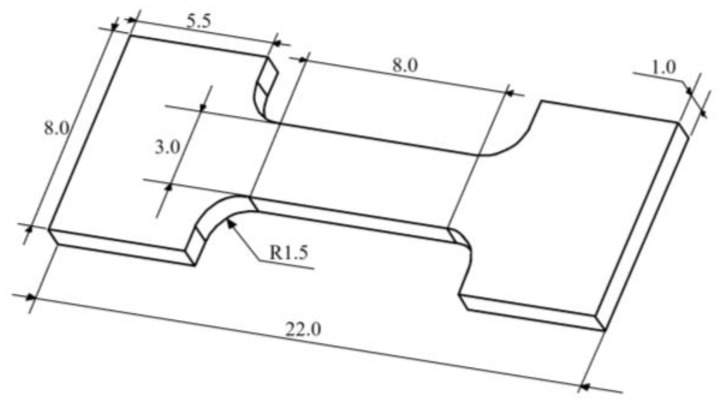
Tensile specimen at room temperature (unit: mm).

The size of the tensile sample is shown in Figure 1, and the rolling direction is selected as the stretching direction. After the sample is processed and formed, it is sanded using sandpaper to ensure that the surface is clean. The tensile test was carried out on the AG-X plus electronic universal material testing machine of the Harbin Institute of Technology (Harbin, China), and the tensile speed was set at 0.5 mm/min. In order to reduce the experimental error, three tensile specimens of each alloy composition were prepared for testing.

### 2.4. Corrosion Behavior Characterization

An electrochemical test was carried out in a CS-350PA electrochemical workstation (Wuhan Corrtest Instrument Co., Ltd., Wuhan, China). The test samples were polished with 1200# metallographic sandpaper, cleaned with alcohol, and dried by blow. A three-electrode system was used for electrochemical testing, with the working electrode as the sample to be tested, the auxiliary electrode as the platinum electrode, and the reference electrode as the saturated calomel. First, an open circuit potential (OCP) test was performed, and the time was set to 1800 s. After the OCP of the sample was stabilized, the electrochemical impedance spectroscopy (EIS) was performed. The EIS was scanned at a frequency of 10^−1^ to 10^5^ Hz, with the amplitude of the potential at 10 mV. Subsequently, the potentiodynamic polarization (PDP) curve was tested. The potential range was set to OCP ± 0.5 V, and the scan rate was 1 mV/s. The corrosion potential (E_corr_) and the corrosion current density (i_corr_) were determined from the cathodic linear region of the polarization curve using Tafel extrapolation. It was tested in Hank’s solution (Na_2_HPO_4_: 0.06; K_2_HPO_4_: 0.06; NaCl: 8.00; KCl: 0.40; MgSO_4_·7H_2_O: 0.20; NaHCO_3_: 0.35; CaCl_2_: 0.14 (g/L)). To ensure the reliability of the results, each experiment was repeated at least three times.

The size of the soaked samples was 10 mm × 10 mm × 1 mm, and the initial weight of the samples was measured using an analytical balance after polishing with 2000# metallographic sandpaper. Hank’s solution was used as the soaking medium. The samples were immersed in a constant-temperature water bath at 37 °C for 7 days, and the solution was changed every 24 h. The ratio of the sample surface area to the solution volume was 1 cm^2^: 30 mL according to ASTM-G31-72 [30]. At the end of the immersion, the surfaces were ultrasonically cleaned to remove corrosion products, blown dry, and reweighed with an analytical balance. Finally, the average weight-loss corrosion rate was calculated according to the formula.

$$CR=\frac{8.76\times 10^{4}\times ∆W}{A\cdot T\cdot D}$$

where CR is the average weight loss corrosion rate (mm/y), ΔW is the mass difference of the sample before and after immersion (g), A is the surface area of the sample (cm^2^), T is the immersion time (h), and D is the alloy density (1.74 g/cm^3^).

## 3. Results and Discussion

### 3.1. Microstructure of Rolled Alloy

**Figure 2:**
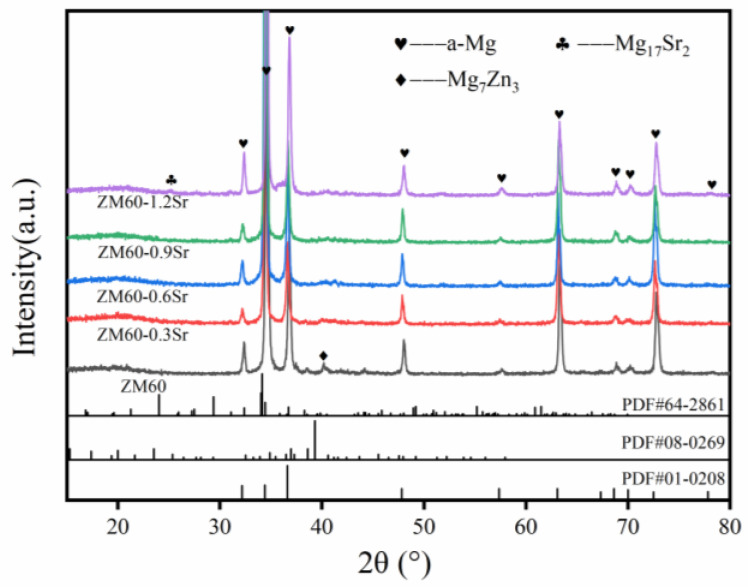
X-ray diffraction patterns of rolled ZM60-xSr.

Figure 2 shows the XRD pattern of the rolled ZM60-xSr alloy. The XRD analysis shows that the phase of the rolled ZM60 alloy is mainly composed of an α-Mg phase and a Mg_7_Zn_3_ phase. After the addition of Sr, the diffraction peak of Mg_7_Zn_3_ was significantly weakened, and a new diffraction peak of Mg_17_Sr_2_ appeared. The peak intensity of the Mg_17_Sr_2_ phase became higher and higher with the increase in Sr content.

**Figure 3:**
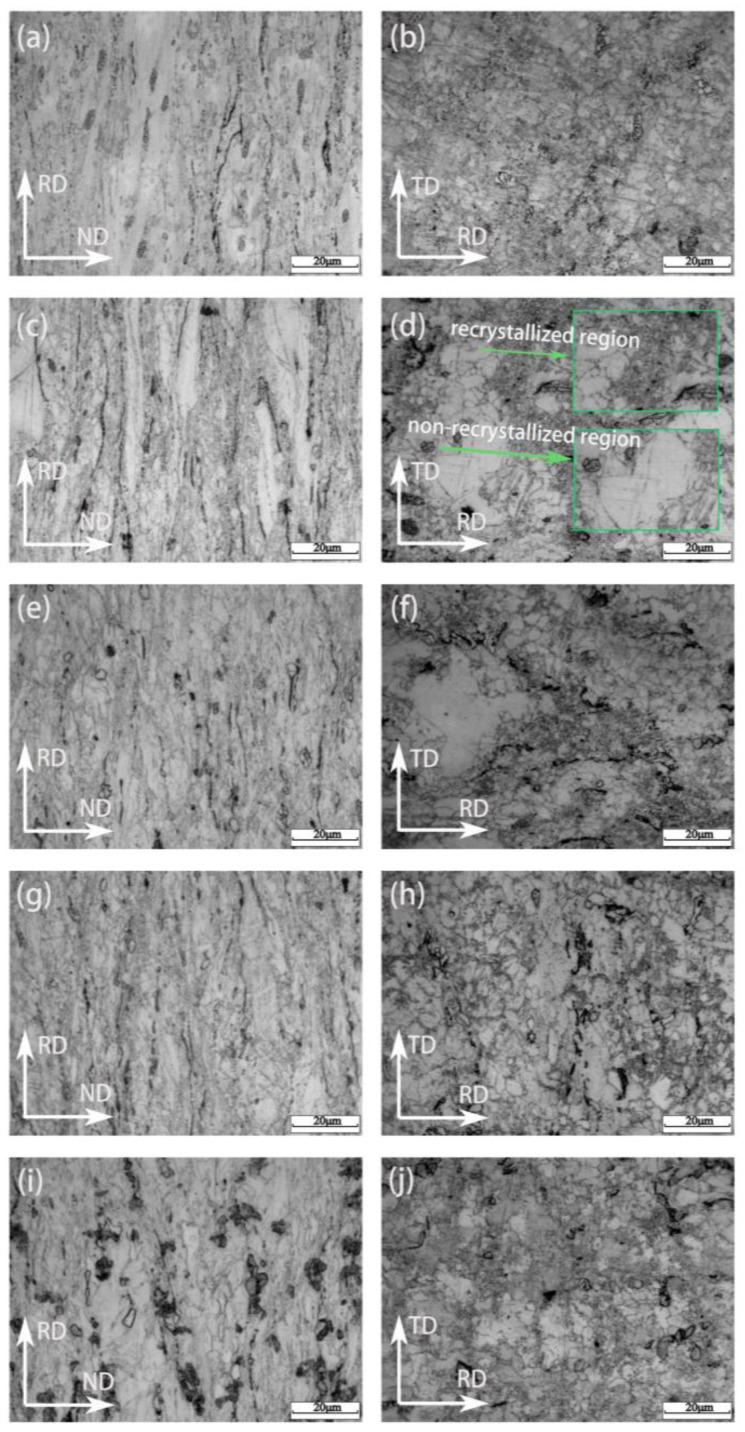
The optical microstructure of RD-ND and RD-TD surfaces of rolled ZM60-xSr: (**a**,**b**) ZM60, (**c**,**d**) ZM60-0.3Sr, (**e**,**f**) ZM60-0.6Sr, (**g**,**h**) ZM60-0.9Sr, (**i**,**j**) ZM60-1.2Sr.

Figure 3 shows the microstructure of the RD-ND surface and the RD-TD surface of the plate obtained by direct rolling of the as-cast alloy. It can be seen from the microstructure of the RD-ND surface of the rolled ZM60-xSr alloy that, under the action of stress, the grain and the second phase are elongated along the rolling direction, accompanied by grain breakage and phase breakage. Figure 3a,b shows the microstructure of the RD-ND surface and the RD-TD surface of the rolled ZM60 alloy, and recrystallization of the rolled ZM60 alloy is observed. Figure 3c,d shows the microstructure of RD-ND and RD-TD surfaces of the rolled ZM60-0.3Sr alloy. It can be found that there are non-recrystallized regions in the microstructure of the alloy, but small fine crystal regions are observed around the second-phase particles and large-size grain boundaries. This is because recrystallization preferentially nucleates around grain boundaries and the second phase, namely particle-stimulated nucleation (PSN) [31]. With the increase in Sr content, the recrystallization of the alloy is more significant. When the Sr content is 0.9 wt.%, as shown in Figure 3g,h, the rolling ZM60-0.9Sr alloy gradually increases due to the amount of second phase with the increase of Sr content, and the second phase hinders the growth of recrystallized grains during recrystallization. The grains in the recrystallized region are fine. When 1.2 wt.%Sr is added, the interface energy of the fine grains is higher and the grain boundary mobility is larger, which leads to the spontaneous “swallowing” of the fine grains in the recrystallized region, which reduces the interface energy and leads to the spontaneous growth of the grains. Therefore, when the content of Sr is less than 0.9 wt.%, Sr inhibits the growth of recrystallized grains of ZM60 alloy. When the content of Sr is greater than 0.9 wt.%, the small recrystallized grains engulf each other, the recrystallized grains grow, and the grain morphology develops towards equiaxed grains.

**Figure 4:**
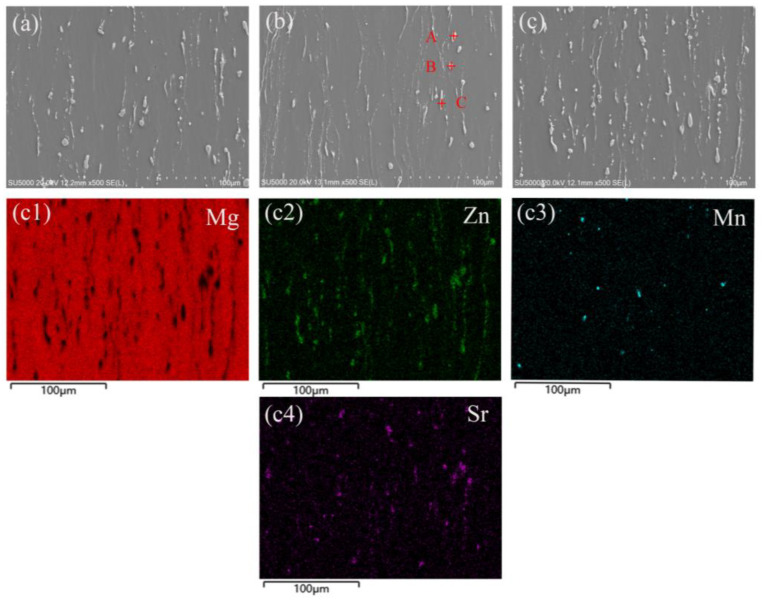
SEM images of RD-TD surface of rolled ZM60-xSr alloy: (**a**) ZM60, (**b**) ZM60-0.3Sr, (**c**) ZM60-0.9Sr, (**c1**–**c4**) EDS analysis of ZM60-0.9Sr alloy.

Figure 4 shows the SEM image of the RD-ND surface of the rolled ZM60-xSr alloy. Figure 4a,c represent the alloy composition as ZM60, ZM60-0.3Sr, and ZM60-0.9Sr, respectively. When the content of Sr is 0.3 wt.%, an EDS analysis of A, B, and C in Figure 4b is carried out, and the results are shown in Table 1. After adding 0.3 wt.%Sr, the second phase can be determined to be Mg_7_Zn_3_ and Mg-Sr phases according to the element content. An EDS energy spectrum analysis of ZM60-0.9Sr alloy was carried out. According to the surface scan results (Figure 4c1–c4), it can be seen that, with the addition of the Sr element, the amount of the second phase in the alloy increased significantly, and the second phase was prolonged and broken along the rolling direction. This may be due to the formation of brittle Mg-Sr second phase [32,33] after the addition of Sr elements to the alloy.

### 3.2. Effect of Sr on Mechanical Properties of Rolled ZM60 Alloy

**Figure 5:**
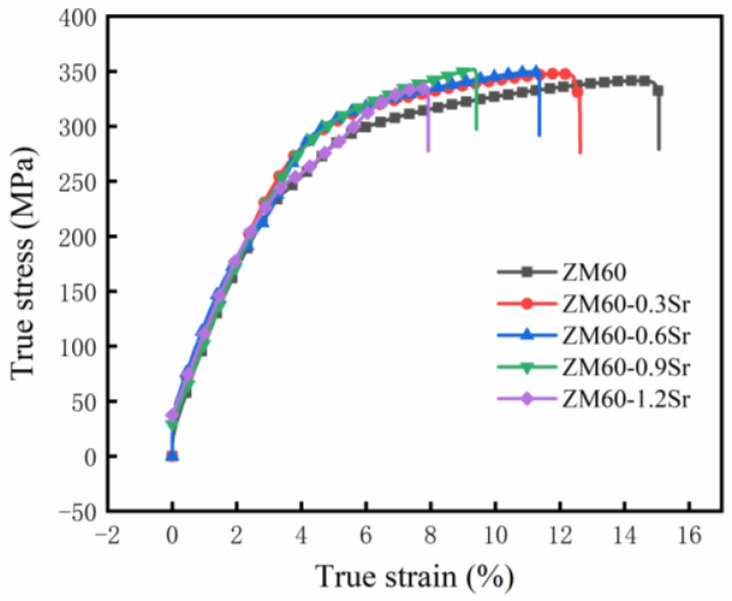
True stress–strain curves of rolled ZM60-xSr alloy.

**Figure 6:**
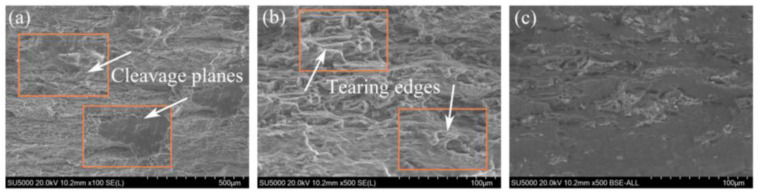
(**a**–**c**) SEM and BSE morphology of rolled ZM60-0.9Sr alloy fracture.

The tensile test of rolled ZM60-xSr alloy was carried out at room temperature. Figure 5 shows the stress–strain curve of rolled ZM60-xSr alloy, and its mechanical parameters are shown in Table 2. With the addition of Sr elements, the strength of ZM60-xSr did not change significantly, and the elongation slightly decreased. Figure 6 shows the SEM and BSE morphology of rolled ZM60-0.9Sr alloy after tensile fracture. There are cleavage planes, dimples, and tearing edges at the fracture surface of the alloy, so it belongs to the quasi-cleavage fracture. It can be found from BSE images that there are a large number of second phases on the fracture surface. When the alloy is subjected to external forces, the second phase will prevent the dislocation slip, and at the same time, the second phase will also play a role in pinning. The second phase is broken in the BSE image of ZM60-0.9Sr. By analyzing the microstructure of the alloy, it is found that Mg_7_Zn_3_ and Mg-Sr phases exist in the alloy because the Sr-containing phase may be Mg_17_Sr_2_, which is a brittle phase, and the increase in the brittle phase will reduce the toughness of the material. In the process of stretching, dislocations will accumulate at the second-phase particles, resulting in an increase in dislocation density at the second-phase particles. Stress concentration will lead to the generation of cracks, resulting in the breakage of the second phase, thus making the magnesium alloy easier to fracture and reducing the elongation.

### 3.3. Effect of Sr on Corrosion Behavior of Rolled ZM60 Alloy

#### 3.3.1. Electrochemical Corrosion Performance

**Figure 7:**
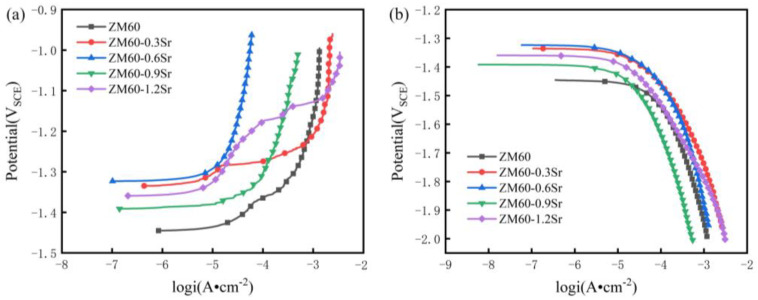
The anodic (**a**) and cathodic (**b**) polarization curves of rolled ZM60-xSr alloy in Hank’s solution.

Figure 7 shows the anodic and cathodic polarization curves of rolled ZM60-xSr alloy in Hank’s solution. The corrosion potential (E_corr_) and corrosion current density (i_corr_) were obtained by the Tafel extrapolation method. First, find out the open circuit potential and linear polarization area (note that there is a cathode and an anode respectively) at the off-circuit potential ±(60–120) mV. Then, put the data points of the two areas into the table for drawing and obtain two segments of nearly straight lines. By linear fitting, obtain the linear equation of the two lines, and the intersection point of the linear equation is the corrosion potential and the corrosion current, as shown in Table 3. The anode region of the potential polarization curve represents the dissolution of the magnesium matrix, and the cathode region represents the hydrogen evolution reaction [34]. From the cathode branch of the rolled alloy, all five components showed hydrogen evolution behavior. In electrochemistry, the corrosion potential represents the tendency of the alloy to self-corrosion in a specific environment. The more positive the potential is, the lower the tendency of the alloy to self-corrosion. When the content of Sr continues to increase, the corrosion potential begins to shift negatively. The corrosion current density represents the corrosion rate in a specific environment, and the higher the corrosion current density, the faster the corrosion rate of the metal in the environment. Compared with ZM60, the corrosion current density did not change significantly when 0.3 wt.%Sr and 0.6 wt.%Sr were added. When the content of Sr continued to increase, the corrosion current density decreased significantly, and the icorr was the lowest when Sr was 0.9 wt.%. Therefore, from the corrosion current density, the ZM60-0.9Sr alloy has the lowest corrosion rate and the best corrosion resistance.

**Figure 8:**
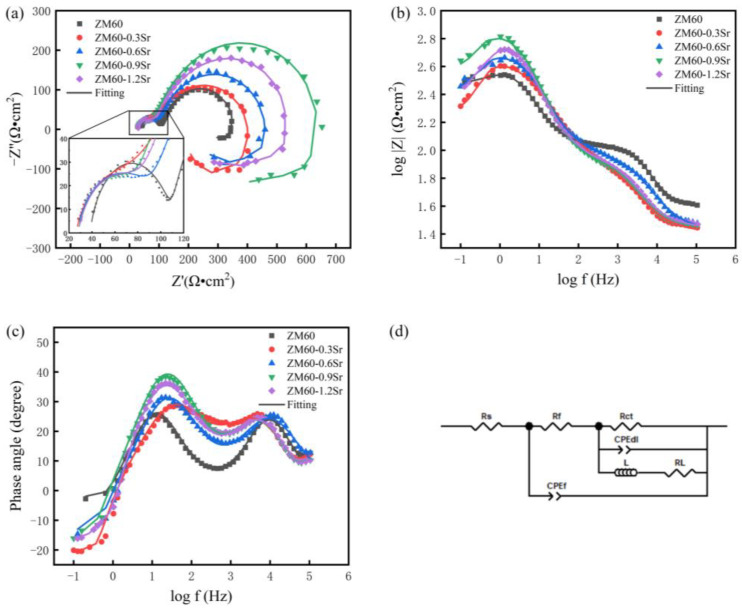
EIS of rolled ZM60-xSr alloy in Hank’s solution: (**a**) Nyquist plots; (**b**) Bode plots of |Z|-frequency; (**c**) Bode plots of phase angle frequency; (**d**) fitted equivalent circuits.

Figure 8 shows the electrochemical impedance (EIS) test of the rolled ZM60-xSr alloy in Hank’s solution. The electrochemical impedance test can reflect the charge transfer impedance of the alloy in Hank’s solution, the electrochemical reaction impedance of the alloy surface, and the impedance of the corrosion product film, which are helpful in understanding the resistance, corrosion mechanism, and corrosion behavior of the alloy. Figure 8a shows the Nyquist plot of the ZM60-xSr alloy. It can be seen that the shape of the Nyquist curve of the ZM60 alloy does not change after adding different contents of Sr. The curve is composed of high- and middle-frequency capacitive arcs in the first quadrant and a low-frequency inductive arc in the fourth quadrant. The capacitive arc in the intermediate-frequency region usually represents the charge transfer process. That is, the transfer of electrons on the electrode surface. The diameter of the semicircle can reflect the size of the charge transfer resistance (R_ct_). Generally speaking, a larger radius of the arc indicates greater resistance and stronger corrosion resistance of the alloy in the test [35]. The intermediate frequency radius of the five alloys is in order from small to large: ZM60 < ZM60-0.3Sr < ZM60-0.6Sr < ZM60-1.2Sr < ZM60-0.9Sr. Figure 8b is the impedance-frequency Bode diagram of the ZM60-xSr alloy. The impedance mode value is an important parameter to evaluate the tolerance performance of the sample. The larger the impedance mode value in the low-frequency region, the stronger the corrosion resistance of the alloy. Figure 8 c, which is the phase-frequency Bode plot, shows two phase peaks at high and intermediate frequencies corresponding to the high- and intermediate-frequency capacitive reactance arcs in the Nyquist plot, respectively. Therefore, the impedance pattern of ZM60-0.9Sr alloy shows better corrosion resistance. In order to further understand the corrosion behavior of the ZM60-xSr alloy through the impedance pattern, the impedance pattern was fitted by Zview 3.3 software. According to the characteristics of the observed impedance pattern, the corresponding equivalent circuit is proposed. By fitting the equivalent circuit pattern to the impedance pattern, the accuracy of the circuit model can be verified, and the electrochemical parameters can be extracted. From the fitting curve (solid line) in Figure 8, it is found that this equivalent circuit diagram can fit the experimental impedance diagram well. The corresponding fitting parameters are shown in Table 4.

The equivalent circuits R_s_, R_f_, R_ct,_ and R_L_ in Figure 8d represent the solution resistance, the spontaneously formed corrosion product film resistance, the charge transfer resistance, and the inductance resistance, respectively. CPE_f_ represents the capacitance of the corrosion product layer on the surface of the sample. CPE_dl_ represents the double-layer capacitance, and L represents the inductance element. The larger value of L indicates that the alloy surface more easily forms a protective film by adsorption [36]. According to the fitting data in Table 4, the R_s_ values of different Sr contents in the alloy are not much different, indicating that the conductivity of the solutions used in the test is similar. The R_ct_ value showed a trend of first increasing and then decreasing and reached the maximum when the Sr content was 0.9 wt.%, indicating that the corrosion product film of the test sample had a great hindrance to the charge migration. At the same time, the inductance L value is also the largest, indicating that the protective film on the surface of the alloy is relatively stable. In conclusion, the impedance profile shows that the ZM60-0.9Sr alloy will produce a more stable corrosion product film compared with the ZM60 alloy during corrosion, which improves the corrosion resistance of the alloy.

#### 3.3.2. Immersion Test

**Figure 9:**
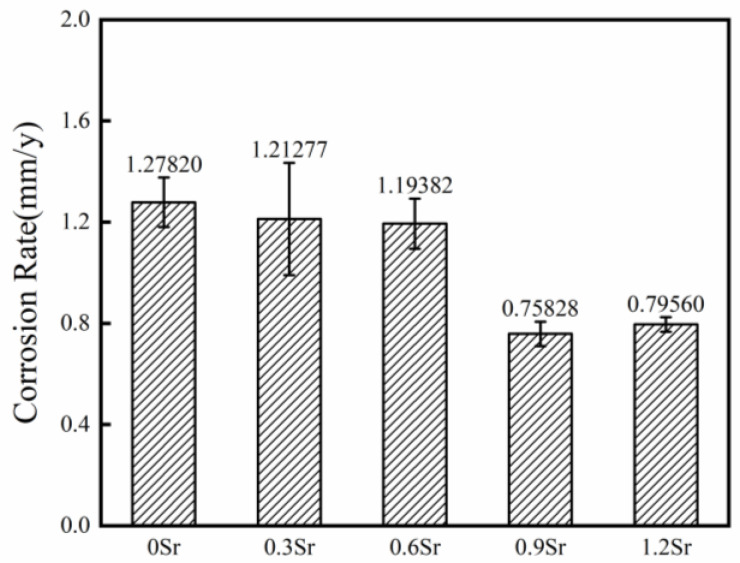
The corrosion rate of rolled ZM60-xSr alloy after immersing in Hank’s solution for 7 days.

Figure 9 shows the average corrosion rate of ZM60-xSr alloy in a rolling state immersed in Hank’s solution for 7 days. The alloy-soaking experiment can intuitively reflect the corrosion resistance of the test alloy. It can be found from the figure that the corrosion rate of the alloy changes little after adding 0.3 wt.%Sr and 0.6 wt.%Sr. When the content of Sr increased to 0.9 wt.% and 1.2 wt.%, the corrosion rate of the alloy decreased significantly. With the increase in Sr content, the grain size and volume fraction of the non-recrystallized region and the recrystallized region are similar, which reduces the galvanic corrosion tendency between the recrystallized region and the non-recrystallized region, thus improving the corrosion resistance. The results of the immersion test and the electrochemical test agree that the alloy with 0.9 wt.%Sr has the best corrosion resistance.

**Figure 10:**
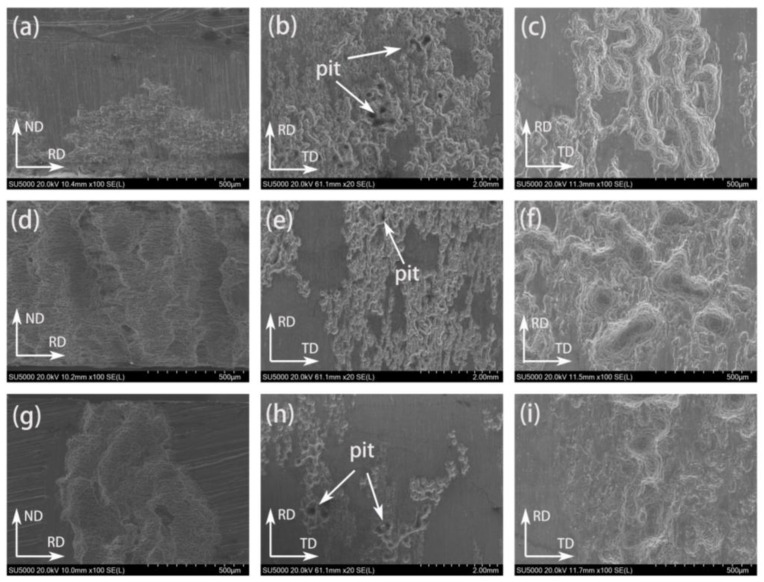
Surface corrosion morphology of rolled ZM60-xSr alloy after removal of corrosion products: (**a**–**c**) ZM60, (**d**–**f**) ZM60-0.3Sr, (**g**–**i**) ZM60-0.9Sr.

Figure 10 shows the corrosion morphology of the rolled ZM60-xSr alloy after soaking in Hank’s solution for 7 days to remove corrosion products on the RD-ND and RD-TD surfaces. Figure 10a,d,g show the corrosion morphology of the RD-ND surface of rolled ZM60, ZM60-0.3Sr, and ZM60-0.9Sr alloys, respectively. It was found that serious exfoliation corrosion marks occurred after adding the Sr element. Figure 10b,e,h are divided into rolling-state ZM60, ZM60-0.3Sr, and ZM60-0.9Sr alloy corrosion morphology from the RD-TD surface. It is found that there are deep corrosion grooves on the alloy surface, and the more serious corrosion places are observed in Figure 10c,f,i. These corrosion gullies were found to be formed by pitting pits connected to each other. With the addition of the Sr element, the corrosion gully of the alloy becomes shallower, the corrosion degree becomes lighter, and the corrosion is more uniform.

**Figure 11:**
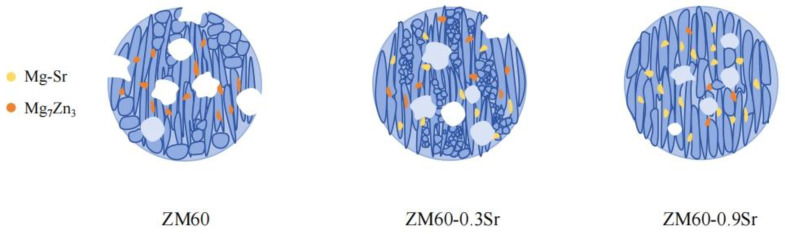
Schematic representation of the corrosion process of rolled ZM60-xSr alloy.

The exfoliation corrosion of ZM60-xSr alloy on the RD-ND surface is mainly caused by local galvanic corrosion. There are two main reasons for galvanic corrosion in ZM60-xSr alloy. First, according to the SEM image of rolled ZM60-xSr alloy in Figure 4, it can be found that the second-phase particles of the alloy after rolling are distributed in a streamline along the rolling direction. These second-phase particles are mainly composed of Mg_7_Zn_3_ and Mg_17_Sr_2_ phases, and the electrode potentials of these phases are higher than those of α-Mg. The lower α-Mg potential acts as an anode, resulting in the preferential corrosion of α-Mg around the second phase. As corrosion continues, the corrosion gradually develops to the interior of the alloy, and the corrosion pit gradually increases. When the adjacent corrosion pit contacts with the alloy, the alloy will have more serious exfoliation corrosion. Second, according to the microstructure of the rolled ZM60-xSr alloy in Figure 3, it is found that there are non-recrystallized regions and fine recrystallized regions in the alloy. Due to the accumulation of a large number of dislocations in the recrystallized regions during the rolling process, corrosion tends to initiate from the recrystallized regions and progress towards the non-recrystallized regions, leading to severe localized corrosion along the fine recrystallized regions and gradually advancing into the non-recrystallized regions [37,38]. When the Sr content is 0.9 wt.%, there are non-recrystallized regions in the alloy, and the grain size volume fraction of the recrystallized regions is similar, which reduces the galvanic corrosion tendency between the recrystallized region and the non-recrystallized region, and improves the corrosion resistance of the ZM60-0.9Sr alloy. According to the corrosion situation, the corrosion mechanism model diagram in Figure 11 was established.

## 4. Conclusions

(1) The addition of the Sr element can affect the recrystallization process of the rolled alloy. When the Sr content reaches 0.9 wt.%, the non-recrystallized large-grain regions are significantly reduced;
(2) After the addition of Sr element, the strength change of the alloy was not significant, and the elongation was reduced;
(3) The corrosion resistance of the ZM60 alloy was significantly improved by adding 0.9 wt.%Sr and 1.2 wt.%Sr, and the corrosion resistance of ZM60-0.9Sr alloy was the best, with an average corrosion rate of 0.75828 mm/y for 7 days.

## Figures and Tables

**Table 1:** Analysis results of rolled ZM60-0.3Sr combined with EDS (wt.%).

Point	Mg	Zn	Mn	Sr
A	43.49	55.03	0.00	1.48
B	43.04	55.43	0.00	1.53
C	52.72	46.64	0.00	0.64

**Table 2:** Mechanical properties of rolled ZM60-xSr alloy at room temperature.

Specimens	σb (MPa)	σ0.2 (MPa)	δ (%)
ZM60	340.93 ± 8.55	212.50 ± 3.26	10.67 ± 2.44
ZM60-0.3Sr	342.19 ± 5.51	219.98 ± 7.11	7.58 ± 1.95
ZM60-0.6Sr	347.69 ± 2.11	220.49 ± 5.74	7.22 ± 1.93
ZM60-0.9Sr	350.04 ± 4.58	221.39 ± 3.50	7.39 ± 2.18
ZM60-1.2Sr	341.21 ± 1.43	208.73 ± 2.16	7.04 ± 2.57

**Table 3:** Fitting results of the potentiodynamic polarization curve of the rolled ZM60-xSr alloy.

Specimens	E_corr_ (V)	i_corr_ (μA·cm^−2^)
ZM60	−1.48 ± 0.02	24.98 ± 0.24
ZM60-0.3Sr	−1.34 ± 0.05	23.56 ± 0.18
ZM60-0.6Sr	−1.32 ± 0.01	22.78 ± 0.12
ZM60-0.9Sr	−1.39 ± 0.05	12.28 ± 0.23
ZM60-1.2Sr	−1.36 ± 0.03	16.76 ± 0.15

**Table 4:** Fitting results of EIS plot of rolled ZM60-xSr alloy in Hank’s solution.

Specimens	ZM60	ZM60-0.3Sr	ZM60-0.6Sr	ZM60-0.9Sr	ZM60-1.2Sr
*R_s_* (Ω·cm^2^)	28.47 ± 0.73	25.76 ± 0.56	26.36 ± 1.21	27.52 ± 1.03	27.57 ± 0.77
*R_f_* (Ω·cm^2^)	71.45 ± 1.04	69.95 ± 1.32	71.43 ± 1.28	72.12 ± 1.13	69.80 ± 1.48
*R_ct_* (Ω·cm^2^)	267.10 ± 7.18	345.20 ± 10.53	409.00 ± 9.33	612.20 ± 4.96	465.20 ± 9.72
CPE*_dl_*-T (F/cm^2^)	(1.81 ± 0.20) × 10^−4^	(1.36 ± 0.14) × 10^−4^	(1.12 ± 0.47) × 10^−4^	(8.63 ± 0.52) × 10^−5^	(8.77 ± 1.12) × 10^−5^
CPE*_dl_*-P (F/cm^2^)	0.83 ± 0.14	0.70 ± 0.03	0.76 ± 0.13	0.79 ± 0.16	0.81 ± 0.12
*L* (Ω·cm^2^)	271.90 ± 8.22	201.00 ± 12.45	236.90 ± 9.71	565.70 ± 22.57	256.00 ± 14.42
*R_L_* (Ω·cm^2^)	772.00 ± 13.38	128.90 ± 5.96	331.70 ± 8.13	579.40 ± 11.72	311.70 ± 2.47
CPE*_f_*-T (F/cm^2^)	(1.53 ± 0.03) × 10^−6^	(1.49 ± 0.05) × 10^−5^	(6.31 ± 0.19) × 10^−6^	(7.81 ± 0.21) × 10^−6^	(1.07 ± 0.06) × 10^−5^
CPE*_f_*-P (F/cm^2^)	0.88 ± 0.06	0.74 ± 0.13	0.76 ± 0.19	0.80 ± 0.02	0.76 ± 0.27

## Data Availability

The original contributions presented in the study are included in the article. Further inquiries can be directed to the corresponding authors.
